# The effect of anion architecture on the lubrication chemistry of phosphonium orthoborate ionic liquids

**DOI:** 10.1038/s41598-021-02763-5

**Published:** 2021-12-15

**Authors:** Bulat Munavirov, Jeffrey J. Black, Faiz Ullah Shah, Johan Leckner, Mark W. Rutland, Jason B. Harper, Sergei Glavatskih

**Affiliations:** 1 System and Component Design, KTH Royal Institute of Technology, 100 44 Stockholm, Sweden; 2 School of Chemistry, University of New South Wales, UNSW Sydney, Sydney, NSW 2052 Australia; 3 Chemistry of Interfaces, Luleå University of Technology, 97 187 Luleå, Sweden; 4 Axel Christiernsson International AB, 44911 Nol, Sweden; 5 Division of Surface and Corrosion Science, KTH Royal Institute of Technology, 100 44 Stockholm, Sweden; 6 Surfaces, Processes and Formulation, RISE Research Institutes of Sweden, 100 44 Stockholm, Sweden; 7 Department of Electromechanical, Systems and Metal Engineering, Ghent University, 9052 Ghent, Belgium

**Keywords:** Mechanical engineering, Mass spectrometry, Ionic liquids, Surface chemistry, Surface assembly

## Abstract

Phosphonium ionic liquids with orthoborate anions have been studied in terms of their interfacial film formation, both physisorbed and sacrificial from chemical breakdown, in sheared contacts of varying harshness. The halogen-free anion architecture was varied through (i) the heteronuclear ring size, (ii) the hybridisation of the constituent atoms, and (iii) the addition of aryl functionalities. Time of Flight-Secondary Ion Mass Spectrometry analysis revealed the extent of sacrificial tribofilm formation allowing the relative stability of the ionic liquids under tribological conditions to be determined and their breakdown mechanisms to be compared to simple thermal decomposition. Overall, ionic liquids outperformed reference oils as lubricants; in some cases, sacrificial films were formed (with anion breakdown a necessary precursor to phosphonium cation decomposition) while in other cases, a protective, self-assembly lubricant layer or hybrid film was formed. The salicylate-based anion was the most chemically stable and decomposed only slightly even under the harshest conditions. It was further found that surface topography influenced the degree of breakdown through enhanced material transport and replenishment. This work thus unveils the relationship between ionic liquid composition and structure, and the ensuing inter- and intra-molecular interactions and chemical stability, and demonstrates the intrinsic tuneability of an ionic liquid lubrication technology.

## Introduction

Ionic liquids are a broad family of compounds based on ionic species that are liquid at accessible temperatures (generally below an arbitrary specific value, such as the boiling point of water^1,2^) though the definition of the term is dynamic^3^. With such fluidity in the definition, it is challenging to identify the first discovery of ionic liquids, though many cite Walden’s preparation of ethylammonium nitrate^4^; in that work, the properties of a salt with a melting temperature of *ca*. 12 °C (a room temperature ionic liquid) are discussed. It was not until the second half of the twentieth century that interest in the subject area increased, catalysed by the potential use of ionic liquid as solvents in electrochemical applications^5^. The areas to which ionic liquid have been directed have expanded, particularly since the development of air stable ionic liquids^5^, to include rather diverse fields from chemistry (including cellulose processing^6^, gas capture^7^, synthesis^8^ and understanding the nature of hydrogen bonding^9^) to more machine industry targeted applications, such as tribology^10^.

The changing technological landscape, with the rapid shift towards vehicle electrification and green energy production being the obvious examples, along with environmental concerns, has resulted in a need for lubricants with enhanced functionality such as electrical responsivity^11–14^. It is the role of chemists to facilitate the tribological advances by developing new formulations, sometimes even with new molecules, in an attempt to feed the industrial and societal demands.

Whilst the range of structures linked by the somewhat arbitrary definition of this class, a key feature is that they are salts and hence consist of ions providing the potential to enhance the affinity of an ionic lubricant towards a surface through electrostatic interactions, and potentially neutralize the adverse effects of tribocharging. There are numerous studies, showing that ionic liquids are competitive with conventional oil-based formulations^15^ in efficiently reducing friction and/or wear.

The chemical reactivity of ionic liquids is, like the broad range of structures, rather diverse and ranges from specifically inert to deliberately functionalised^16^. The latter leads to the potential for targeted design in tribology through consideration of the chemical affinity of the constituents of the ionic liquid lubricant towards the surfaces in contact. There is the opportunity to incorporate chemical functionalities that might improve the tribological performance through tailored surface interactions. The use of ionic liquids for contact lubrication imposes some restrictions, however. A lubricant is usually operated under quite harsh conditions (gigapascal range pressures, contact temperatures above 100 °C, *etc*.). These conditions mean that any ionic liquid used as a neat lubricant would be required to have high chemical stability. Another important point to consider surrounds potential impurities in the ionic liquids. The presence of halogen atoms, such as chlorine and fluorine, which the vast majority of commercial ionic liquids contain, is untenable in tribological applications due to issues such as corrosion^17,18^. With the above argument in mind, there have been a growing number of studies where authors test non-halogenated ionic liquids as prospective lubricant and additives^15^.

While the number of possible ionic liquids is enormous based solely on potential ion combinations (estimates range from *ca*. 10^6^^19^ to *ca*. 10^12^^20^), phosphonium based ionic liquids stand apart, especially in terms of prospective tribological applications. Particularly, they have been noted to be more thermally stable than ionic liquids based on quaternised nitrogen centres^21^. Further, their degradation products (phosphanes, phosphines, phosphoranes and alkanes^22^, and ultimately phosphate) are not as chemically reactive as, for example, the carbenes suggested to be formed during the decomposition of imidazolium-based ionic liquids^23^. Phosphonium ionic liquids are also readily prepared on the industrial scale^24^. Based on these features, there are already numerous reports showing that phosphonium-based ionic liquids perform well both as lubricants and as lubricant additives^25–31^. Notably, several groups have reported the formation of phosphorus-containing structures in the worn areas of the surfaces lubricated with neat phosphonium-containing ionic liquids and relate these structures to the improvement of tribological properties^26,30^. However, it is important to note that in those cases the ionic liquids involved phosphate and phosphinate anions (in conjunction with the phosphonium cation).

**Figure 1:**
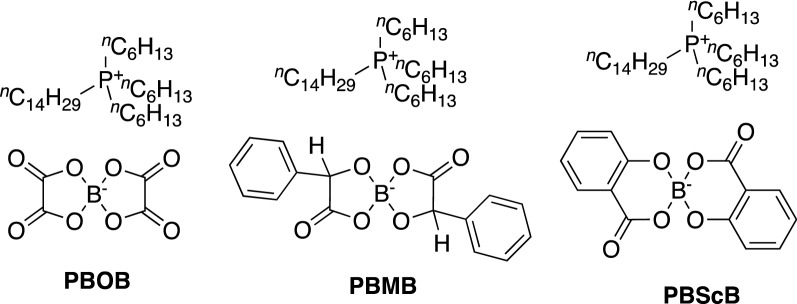
The ionic liquids used in this work: trihexyl(tetradecyl)phosphonium *bis*(oxalato)borate (PBOB), trihexyl(tetradecyl)phosphonium *bis*(mandelato)borate (PBMB), and trihexyl(tetradecyl)phosphonium *bis*(salicylato)borate (PBScB).

Currently lacking is an understanding of (i) the manner in which phosphonium ionic liquids facilitate lubrication, (ii) the mechanism of their breakdown, and, particularly, (iii) the relationship of the structure of the ionic liquid components to both of the above. On point (i), both traditional sacrificial tribofilms^32^ and ionic boundary layers^33^ have been invoked to explain the tribological properties of ionic liquids. Where referred to, breakdown of ionic liquids has focussed upon the cation of the ionic liquid and the resulting decomposition products. As such, the work described herein considers the tribological properties of three rationally chosen ionic liquids (Fig. 1) with a common cation but different anions, in order to evaluate the role of the anion (and its structure) in tribofilm formation and lubrication performance.

The trihexyl(tetradecyl)phosphonium cation was chosen due to it significant stability and preliminary data demonstrating the lubricity of an ionic liquid based on this cation^33^. The three anions were based on boron centres; boron-based compounds are well recognized by the tribological community for reducing wear and friction^34–36^. The selected orthoborate structures ensure that the compounds are liquid at room temperature (*cf.* many tribologically beneficial compounds are solids at room temperature^37,38^, complicating their application). The ligands on the boron were chosen to vary key features, including boron heteronuclear ring size, flexibility of the components on the ligand, and the presence of an aromatic system. The latter feature was inspired by tricresylphosphate antiwear additives^39^, with phenyl groups having been shown to affect the lubrication performance of ionic liquids in steel-on-aluminium contacts^40^; the lowest wear was attributed to the ionic liquid with flexibly attached aromatic rings, while the lowest overall friction was reported for the ionic liquid with no aromatic groups. These features are likely due to changes in surface interactions on introducing the phenyl rings.

Whilst this previous study^40^ implies an importance of the phenyl substituent in the anion, it was focused on the physical and chemical properties of bulk liquids and thus lacks any investigation of either the lubricated surfaces, the effect of changing temperature or the molecular mechanisms underlying the lubrication properties of ionic liquids. Thus, in this study we are investigating the tribological performance of the neat ionic liquids, upon lubricating industrial grade bearing steel surfaces. Along with the effects on lubrication of changing the anion, the stability of the ionic liquids and the importance of their breakdown products was considered. Subsequently, thermal effects and the influence of surface topography on ionic liquid lubrication and breakdown were considered.

### Effects of ionic liquid structure on lubrication and tribofilm formation

**Figure 2:**
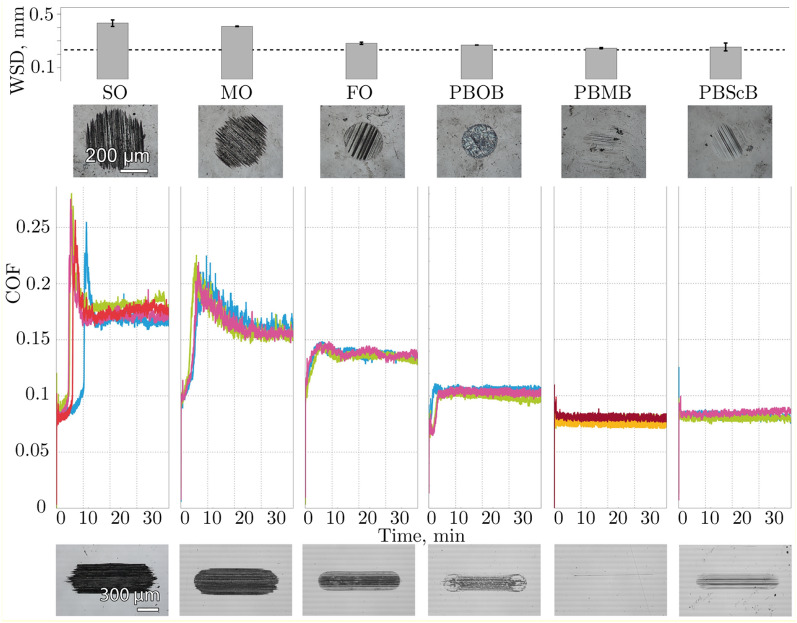
Ball wear scar diameters (WSDs), friction traces and optical images of the wear scars on the ball and PD surfaces for constant load tests involving the lubricants shown. Different coloured traces represent different replicate experiments. The dashed line in the WSD plot indicates the initial Hertzian contact diameter.

Initially the tribological tests were carried out using polished disks (PD) as the plate component of the experiment, to maximise the uniformity of any tribofilm formed and to minimise any effects of surface topography on lubricant decomposition. The time evolution of the coefficients of friction (COF) obtained in the constant load tests using PD plates is presented in Fig. 2, along with analysis and images of the wear scars on the ball and the PD surfaces. Note that a series of base oils (synthetic oil, SO; mineral oil, MO; formulated oil, FO; for details see “Methods”) have been used as a comparison.

An immediate observation from this Figure is that both base oils, MO and SO, follow approximately the same pattern of friction over time in each of the three respective tests, despite there being a significant difference in viscosity (see “Methods”). The initial value of the COF was around 0.1 or even lower, but within 5 min it increased rapidly up to values of 0.2 and above before eventually declining and plateauing at values of *ca*. 0.17 and *ca*. 0.15 for the SO and MO, respectively. Such behaviour is not uncommon and is readily explained. At the beginning of the tests, the surfaces were separated with an initially adsorbed layer of lubricant, however, as the test proceeds, the lubricant layer is removed and the system enters an adhesive wear dominated boundary lubrication regime (which explains the observed high, yet stable, friction). In comparison, the fully formulated oil FO demonstrates a lower COF plateau value of *ca.* 0.14 with no pronounced adhesion spike. This lower value reflects the presence of antiwear additives such as zinc dialkyldithiophosphates (ZDDPs)^32^; this additive also provides a degree of wear protection as observed in the smaller, more well-defined wear scar. It thus appears that, under these test conditions, lubricant viscosity is of much less significance than the chemical composition of the lubricant for these systems.

Also immediately apparent, is that each of the ionic liquids notably outperform all of the reference oils in terms of friction behaviour: the COF values are much lower (PBMB and PBScB *ca*. 0.08; PBOB slightly higher at *ca.* 0.1) and the COF distributions are narrower, with better signal to noise ratio. The narrow COF distribution indicates that the tests were performed within a stable lubrication regime. At the same time, the COF values are much lower than expected for adhesive wear. In contrast to the pure oils, the friction coefficients are now, albeit weakly, systematic with the viscosity of the liquids, which implies either a larger surface separation, a lower roughness, or both. It is thus reasonable to infer that the conditions most probably correspond to the boundary lubrication regime with the surfaces separated by boundary films^33^; the nature of these films will be discussed subsequently.

The low friction and the absence of the initial adhesion spike correlates well with the apparent wear protective properties of the ionic liquids. It is apparent that they all demonstrate a performance on the same level as the formulated oil with wear scar diameters on the balls around half that of the SO case. The particulate scratching is also markedly less pronounced and in the case of the [BMB]^-^ anion, the area is actually overestimated by the assumption of a circular scar. On these flat surfaces the wear is also much less pronounced even than for the FO, and in the case of PBMB a wear scar is almost not visible.

While the differences between the ionic liquid friction coefficients are small, they are highly reproducible, and the wear scars on both the balls and the flat PD surfaces demonstrate that there are differences in their tribological behaviour. This difference in the coefficient of friction clearly indicates that changing the anionic component of the ionic liquid affects their lubricating ability; particularly, the presence of an extended organic structure including aryl rings (PBMB and PBScB) decreases the coefficient of friction, without the five minute rise to a plateau region, and results in more homogenous wear scars. The differences may be as a result of changes in the electronic distribution in the anions (including the presence of aromatic systems, which introduce interactions with the surface—polar–*π* interactions^41–43^) though the propensity to breakdown under the conditions of the experiment must also be taken into account.

In order to investigate the robustness of the lubricating behaviour of the ionic liquids, tests involving stepwise increase of the load (up to 400 N) were carried out. Once again, the ionic liquids outperformed the unformulated oils and showed even more stable friction response than the formulated oil (for full discussion, see Supporting Information).

Scanning electron microscopy (SEM) of the worn PD surfaces was utilised to determine if there were any changes in the surface composition and structure during the experiments (see Supporting Information). Only wear scratches, parallel to the direction of movement, are seen in the case of PBMB and PBScB, whilst a patterned structure on top of these scratches is seen in the case of PBOB likely composed of a tribofilm consisting of lighter elements. The elemental composition of the structures formed in this case was examined using Energy-dispersive X-ray spectroscopy (EDS; see Supporting Information) which confirmed increases in the incidence of carbon and oxygen in the wear scar and a depletion in the levels of iron and chromium, consistent with an overlayer of a more organic nature. Importantly, however, this methodology did not provide any detail concerning either boron or phosphorus (as the sensitivity is insufficient to detect these elements at the levels present in the tribofilms), which would be characteristic of breakdown of the anion and the cation, respectively. Whilst the presence of organic species in one case and not the others implies that the anion is breaking down and that the structure of the anion determines that breakdown, it is not possible to determine whether this breakdown is due solely to the presence of the [BOB]^-^ anion or whether there are reactions involving the cation also. This point is sufficiently important to justify further detailed analysis of the tribofilms.

Time of Flight-Secondary Ion Mass Spectrometry (ToF–SIMS) analysis was thus carried out on the wear scars produced above using each of the ionic liquids. Immediately apparent were signals consistent with both carbon and oxygen being present on the surface, confirming the results from the EDS experiments (see Supporting Information). This methodology also allowed identification of signals due to boron and phosphorus containing species in the tribofilms, indicating that both the cation and anion have been broken down to some extent during the process.

**Figure 3:**
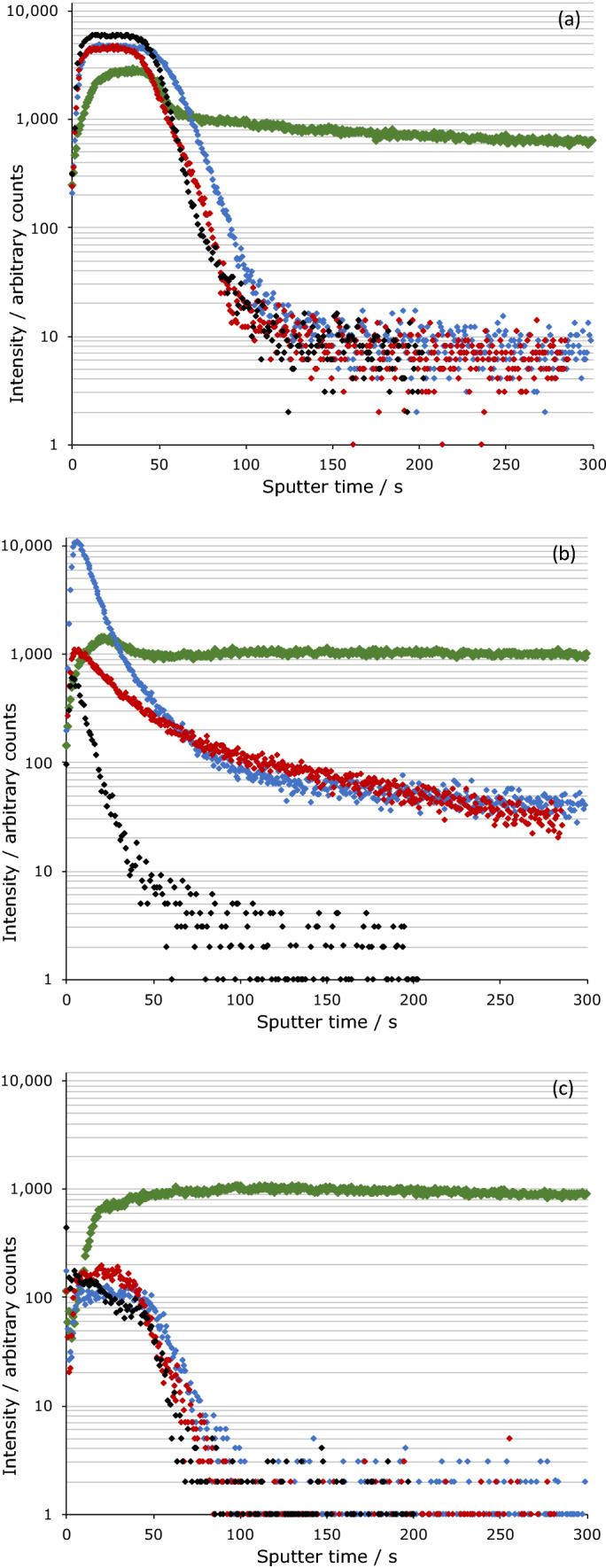
ToF–SIMS depth profiles for the PD surfaces, showing the change in ion intensity over time for **(a)** ^54^FeO_2_^−^, **(b)** BO^−^ and **(c)** Fe_2_B_3_O_3_^−^. The colours correspond to the ionic liquid used PBOB (green), PBMB (blue) and PBScB (red), with the untreated surfaces shown separately (black).

Representative sputtering time profiles are shown in Fig. 3; these are typically referred to as ‘depth profiles’ (with sputter time being an indirect measurement of depth into the sample) as longer sputtering times expose materials from further down the sample and large sputter times correspond to bulk composition. The PBOB case resulted in significant oxidation of the steel surface, with *ca*. 10–200 times the abundance of Fe_x_O_y_ species compared to the untreated steel surface (see Fig. 3a, noting that ^54^FeO_2_^-^ was selected in preference to the more abundant ^56^FeO_2_^-^ as the ^56^FeO_2_^-^ signal saturated the detector under experimental conditions). This oxide development was not as pronounced for the other ionic liquids studied, with the PBScB case showing no significant change in the Fe_x_O_y_ signals relative to the steel surface, and the PBMB case only having a small change of the initial oxide layer, persisting for *ca.* 1.2 times as long as the steel control. Similar trends to those seen for the iron species were observed for the O^-^, O_2_^-^ and OH^-^ ions (see Supporting Information).

All of the samples, including the steel control, displayed high levels of boron and oxygen containing species (such as BO^-^ and BO_2_^-^, the former is shown in Fig. 3b) directly at the surface. Importantly, for the steel control the signal quickly dropped to levels too low for the instrument to reliably detect, consistent with the bulk composition of the steel^44^. For the PBOB case, high signal levels were sustained deep into the tribofilm sample, indicating an abundance of boron containing species. The PBScB and PBMB cases displayed intermediate behaviour; with the signals falling off more slowly than the steel control case; whilst the build-up of boron and oxygen containing species was not as marked as in the PBOB case, it still occurred. Of note is that in the PBMB case, the levels of boron and oxygen containing species at the surface were higher than in any other sample. This abundance may indicate the presence of a thin, irreversibly bound, sacrificial boundary film.

The only source of the boron containing species in the tribofilm is the anion of the ionic liquid used. The relative amounts of the species present are consistent with either (i) the PBOB ionic liquid breaking down to a greater extent than the PBScB and PBMB ionic liquids or (ii) the breakdown products of the PBScB and PBMB not being as readily detectable. It is reasonable to assume that the former is more likely, based on expected chemical products, TGA measurements for the same systematic anionic series^45,46^, and observations of tribofilm formation; this will be discussed further below.

It is of note that the PBOB steel sample also displayed a significant Fe_2_B_2_O_3_^−^ ion signal, whilst the other ionic liquid cases gave results indistinguishable from the parent steel sample (Fig. 3c). This observation, coupled with the greater extent of oxidation of the iron surface in that system suggests that the anion of the ionic liquid may be being broken down either at, or by, the iron surface. Irrespective, the greater breakdown of anion in that case leads to greater oxidation of the iron. Note that while both PBScB and PBMB samples show significant degradation of the anion (as illustrated by the BO^-^ and BO_2_^-^ data) they do not display the associated oxidation of the iron surface. However, those observations are consistent with the PBMB degrading more than the PBScB sample.

The phosphorous signals behaved in a similar fashion to those due to the boron containing species (see Supporting Information). At the surface there was a large number of species containing phosphorus and oxygen (e.g*.* PO_2_^-^ and PO_3_^-^). For the steel sample, these signals quickly dropped to levels too low to reliably detect, while the ionic liquid samples had a slower decrease with sputtering time and never reached the same low levels. The abundance of these signals was greatest for the [BOB]^-^ based ionic liquid sample followed by PBMB then PBScB.

While discussing the phosphorus-based signals, it is important to note two points: that the most likely source of the phosphorus is the cation of the ionic liquids, and that all of the ionic liquids considered here have the same cation. Hence, the fact that the phosphorus signals differ between samples shows that the cation did not break down to the same extent in each sample; this outcome demonstrates that the ions of the ionic liquid cannot be considered in isolation. The greater formation of oxidised phosphorus species in the PBOB case compared to the others considered suggests that the process of breakdown of the [BOB]^−^ anion favours (oxidative) breakdown of the cation and/or retention of the oxidised species.

The analysis of other signals in the ToF–SIMS data provides less differentiation between the samples than those cases introduced above. For example, the signals containing carbon showed similar behaviour for the PBMB and PBScB cases, whilst the PBOB case showed greater abundance for carbon-based signals throughout the sample (see Supporting Information). These data simply reinforce the argument presented above, and suggest that the PBOB sample does break down more than the other ionic liquids during the lubrication experiment. Thus, it seems that only PBOB is prone to a significant degree of oxidative breakdown and subsequent tribofilm formation.

### Analysis of the breakdown of the ionic liquids used and explanation of tribofilm formation

The argument presented above relies on the breakdown of ionic liquids to generate species that form a tribofilm in the PBOB case. As such, it is of interest to analyse (using the same ToF–SIMS methodology) pristine samples of the ionic liquids, samples that had been heat treated (to promote breakdown, see subsequent arguement), and samples of the ionic liquid that had been used as lubricants in the tribology experiments. The spectra were analysed based on predicted breakdown products (see structural analyses in the Supporting Information, which builds on the work of Binnemans^22^) and changes in ion abundance relative to the pristine ionic liquid considered. Heat treatment was carried out simply using a heat gun. Whilst other methods, such as thermogravimetric analysis coupled with an analytical method to detect breakdown products, could be used to examine breakdown of the ionic liquid, the key point here was to provide a straightforward method to generate broken down ionic liquid for ToF–SIMS analysis.

The most significant changes to the spectra were seen for the PBOB ionic liquid (see Fig. S8). In the spectra for the ionic liquid used in lubrication experiments, reduction (relative to the pristine sample) of the signals corresponding to the [BOB]^-^ anion and the C_2_BO_5_^-^ signal were observed, consistent with significant breakdown of the anion during the lubrication experiments. For the heated systems, the absolute size of the signal corresponding to the [BOB]^-^ anion decreased significantly, after only a short (10 s) heating period, and relatively, the breakdown products of the anion (C_2_B and C_0_B species) were more abundant than the parent anion. No signals due to the anion were observed in the sample heated for 60 s. These outcomes are consistent with observations from the heating experiments, where significant evolution of gas occurs initially but stops prior to the end of the 60 s heating period, along with changes to the NMR spectra indicating multiple boron species (see Supporting Information for ^11^B and other key NMR analyses). These data indicate the breakdown of the [BOB]^−^ anion, both in the lubrication samples and on heating. Based on the signals seen, it is reasonable to suggest that it occurs by the mechanism proposed in the Supporting Information, noting that it is likely that either some of the breakdown products are lost prior to analysis (as they are volatile) or are not observed in the negative ion mode.

**Figure 4:**
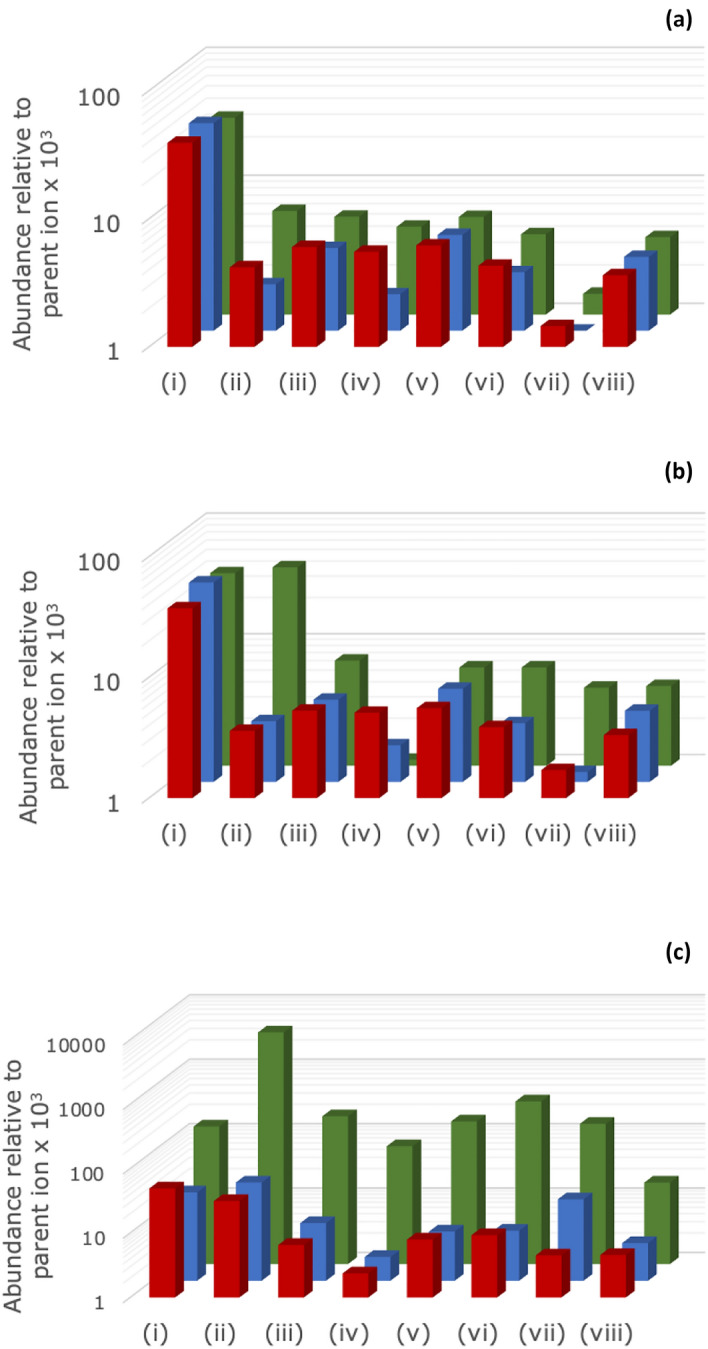
Relative abundance of selected ions in the ToF–SIMS analysis of the **(a)** pristine ionic liquid, **(b)** ionic liquid sample heated for *ca*. 10 s, and **(c)** ionic liquid sample heated for *ca*. 60 s. The colours correspond to the ionic liquid: PBOB (green), PBMB (blue) and PBScB (red). The columns represent (i) C_26_H_56_P^+^, (ii) C_26_H_56_PO^+^, (iii) C_12_H_28_P^+^, (iv) C_18_H_40_PO^+^, (v) C_6_H_16_P^+^, (vi) C_14_H_30_PO^+^, (vii) C_6_H_14_PO^+^ and (viii) C_6_H_16_PO^+^.

Whilst analysis of samples in negative ion mode provided data on the anion breakdown, the corresponding ToF–SIMS positive ion mode spectra provide data on the presence of phosphonium cations (and the like) hence giving an indication on the extent of cation breakdown (Fig. 4). Whilst limited changes were seen in the positive ion mode spectra of the PBOB sample after being used for lubrication, for the sample heated for 60 s, the signals corresponding to the loss of alkyl chains (including loss of both C_6_ and C_14_ chains) and oxygenation of the phosphorus centre were seen to increase. Likewise, the proportion of the final breakdown product (phosphate) increases upon heating. Whilst no phosphorus signals other than phosphate were significant in the tribofilm, these data support the breakdown pathways proposed (see Supporting Information) and are consistent with NMR data showing multiple phosphorus signals in those samples (see Supporting Information). Overall, these arguments demonstrate that the anion of the PBOB ionic liquid breaks down first, and the breakdown of the anion leads to the breakdown of the cation.

The PBMB samples (see Figure S9) only showed significant breakdown of the anion upon heating for 60 s, with the [BMB]^-^ anion still present, but with a lower abundance, as well as signals for borate compounds and the corresponding alcohol. (The sample heated for 10 s showed no signs of significant breakdown.) The lubrication test sample of the same ionic liquid showed greater decomposition of the anion than for either of the heated sample, with no significant signal due to parent anion. Importantly, no breakdown of the cation was observed in any of these cases.

The difference in apparent stability of the [BOB]^-^ and [BMB]^-^ anions during tribological tests can be explained by PBOB forming a sacrificial tribofilm while PBMB does not; as PBOB breaks down during tribological tests, these breakdown products remain on the steel surface; conversely as PBMB breaks down, the breakdown products instead partition into the liquid. That is, the analyses of the surfaces and the lubricant after use in the tribological tests provide complementary information. The lack of observable sacrificial tribofilm in the PBMB case thus supports the argument for a lubricating ionic boundary layer. This behaviour is akin to that seen for PBOB under different conditions^33^, noting that the presence of an aryl group will introduce additional interfacial self-assembly constraints.

The PBScB samples (see Fig. S10) displayed the greatest chemical stability of the ionic liquids tested. There was no observed breakdown of the cation of the ionic liquid, and there was very little change in intensity for the signal corresponding to the [BScB]^-^ anion upon either heating or use in the lubrication tests. While there were some changes in intensity of daughter ions on heating, the key point is that there was no significant breakdown of the [BScB]^-^ anion. This lack of breakdown implies that a sacrificial tribofilm is not responsible for the observed friction reduction, rather a lubricating boundary layer (again incorporating interactions involving a aryl group) is likely key.

As a summary, these ToF–SIMS analyses of the ionic liquids demonstrate that:

- the relative chemical stability of the systems is PBOB < PBMB < PBScB (and this is consistent with other available data – NMR (see Figs. S11–22), TGA^45,46^),
- the mechanisms proposed for the breakdown of the ionic liquid are reasonable and likely occur under lubricating conditions, and
- the extent of breakdown of the cation of the ionic liquid is dependent on the anion present.

**Scheme 1:**
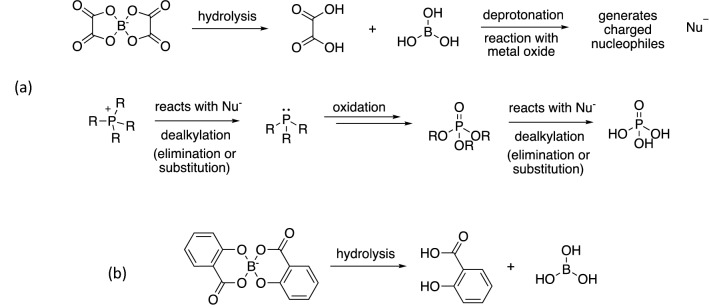
**(a)** Stepwise breakdown of PBOB (for full mechanistic analysis, see Supporting Information). Breakdown of the anion generates the nucleophiles (shown as Nu^−^) required for subsequent breakdown of the cation, which is shown with generic R groups rather than specifying specific alkyl chain lengths. The order of the multiple oxidation steps and the final dealkylations may vary but the outcome is the same. **(b)** The breakdown of the *bis*(salicylato)borate anion to give salicylic acid and boric acid.

These observations allow a stepwise breakdown of the ionic liquids to be proposed, which is exemplified in Scheme 1a which shows the PBOB case (chosen because it is shown to breakdown most effectively, but the equivalent can be generated for the other ionic liquids). This argument shows the key steps that are important in the breakdown of the ionic liquid (anion breakdown and hence nucleophile generation, dealkylation of the phosphorus centre, oxidation of the phosphorus centre). It is important to emphasise that the first step of the process is the breakdown of the anion and the subsequent breakdown of the cation is thus dependent on the products of that first step. As a result, the anion architecture is key in determining the tribochemical behaviour of the ionic liquids. It is important to note that the breakdown of the anion requires water and hence water content of the ionic liquid is important; this has been demonstrated both in the chemical reactivity of the ionic liquid anion^33^ and the formation of tribolayers^47^. (For details of water content, see “Methods” section.) The results here demonstrate that at the water contents used, the ionic liquids are kinetically stable at room temperature but undergo hydrolysis at high temperatures, such as in a tribological contacts.

It is worth considering the origin of the difference in stability of the anions. Initially considered were relative bond strengths of the B-O bonds (based on the daughter ions observed in the ToF–SIMS analysis). These bond strengths might be estimated by the acidity of the corresponding C–OH systems^48^. Those values, however, do not follow the same trend as the observed changes in stability. An alternative proposition is the heteronuclear ring size in the anion; the difference in stability might be related to the bond angles (and hence ring strain) in these systems. The larger ring size in [BScB]^−^ would be expected to stabilise that anion relative to [BMB]^−^, where the five membered ring would be expected to introduce some ring strain, particularly due to the presence of an *sp*^2^ hybridised carbon centre. This problem would, in turn, be more marked for the [BOB]^−^ case where there are more *sp*^2^ hybridised centres; the O–B–O bond angle is noticeably constrained in that case. With this in mind, it appears that ring strain dominates the chemical stability of these systems.

### The effect of surface topography on ionic liquid breakdown and lubrication

**Figure 5:**
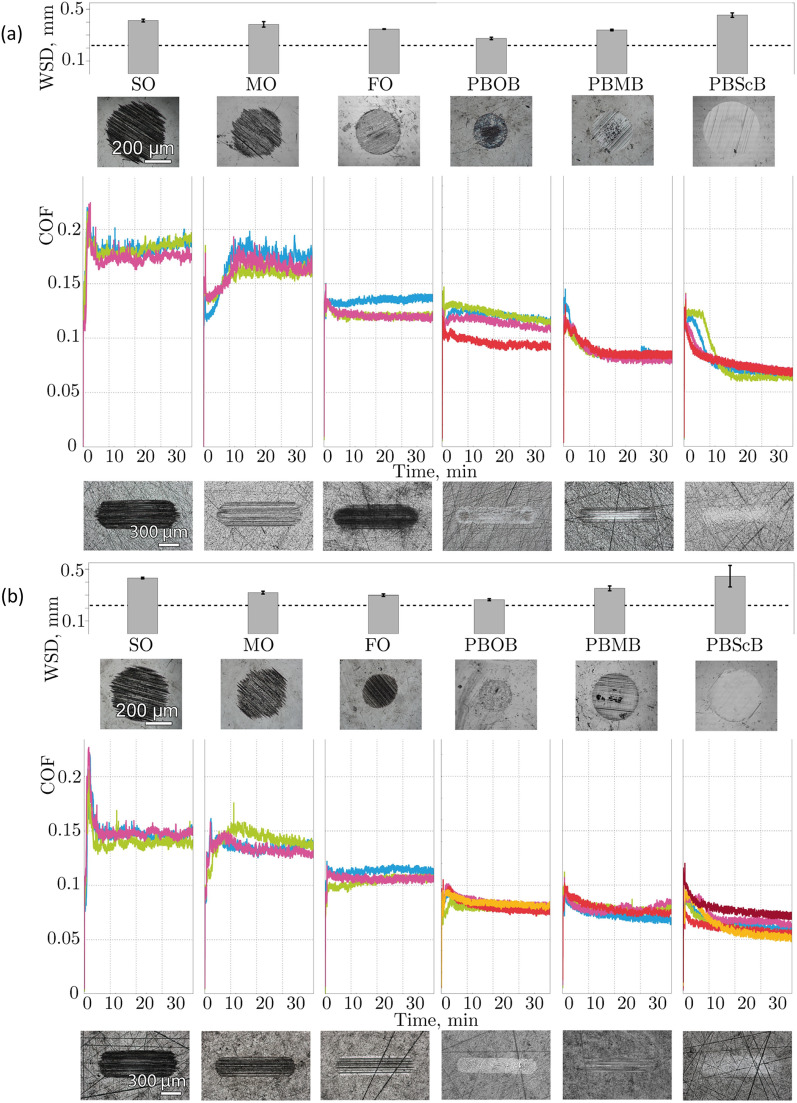
Ball wear scar diameters (WSDs), friction traces and optical images of the wear scars on the ball and either **(a)** SD surfaces or **(b)** BW surfaces for constant load tests involving the lubricants shown. Different coloured traces represent different replicate experiments. The dashed lines in the WSD plot indicate the initial Hertzian contact diameter.

Rougher surfaces may also contribute to, and potentially change, the decomposition processes of ionic liquids under lubricating conditions due to an increased severity of surface asperity interactions. More industrially relevant surfaces with higher roughness values (Standard SRV disks (SD) and portions of needle bearing washers (BW)) have been studied as the flat surfaces in constant load tests. Once again, the time evolution of the coefficients of friction (COF) obtained in the constant load tests using these plates is presented as Fig. 5, along with analysis and images of the wear scars on the ball, and the SD and BW surfaces. As was observed in the case of the PD surfaces, comparison of the results for the unformulated reference oils indicates no noticeable influence of the lubricant viscosity on the COF, once again implicating adhesive wear dominated boundary lubrication.

The surface topography has a significant effect on the values of observed COF. This effect is particularly apparent on moving to the BW surfaces (Fig. 5b) where a combination of reduced real area of contact and enhanced lubricant supply via the scratches and channels^49,50^ (*ca.* 1 μm deep, see Supporting Information, Fig. S1) lead to a reduced COF.

Once again, the ionic liquids provide a significantly improved lubricity compared to the unformulated reference oils on both the SD and BW surfaces, however, the individual shapes of the COF evolution curves are now somewhat different. Rather than the essentially flat behaviour observed for ionic liquids in Fig. 2, the ionic liquids now show a behaviour similar in form to the reference oils, with an initial increase in COF followed by a slower decrease to a plateau, albeit at much lower values of the coefficient of friction. Such profiles would suggest a certain amount of adhesive contact friction is being displayed and thus that the boundary films are not as successful at preventing wear in the case of the rougher surfaces. This argument is supported by the wear data, with the scars much more clearly manifested in Fig. 5 than in Fig. 2. The severity of the scarring appears to be lower than for the reference oils, but the wear scar diameter is comparable.

PBScB shows similar, extremely limited sacrificial tribofilm forming behaviour on these surfaces, as was observed for the PD surfaces. After an initial drop, the final value of the COF is below that observed for the PD surface. This decrease is likely due to extra lubricant that is trapped in the surface scratches^49,50^. PBMB behaviour also follows the same pattern, with reduced friction compared to the reference oils and minimal sacrificial tribofilm formation.

PBOB shows the most complicated behaviour with COF values notably different for the SD and BW surfaces. For SD surfaces, friction tends towards the values observed for the PD surfaces, which would suggest that the surfaces are being polished throughout the test. At the same time for BW surfaces, the resultant COF values are significantly lower than for the other surfaces. Moreover, the wear scar images show significant sacrificial tribofilm formation on PD and SD surfaces, but negligible sacrificial tribofilm formation on BW surfaces. This decreased amount of sacrificial film formation (likely due to the breakdown of the ionic liquid as argued above) may be result of a replenishment process. At a particular point when the ball passes over a scratch, exposing itself to a reservoir of lubricant, the lubricant residing in the scratch experiences a force, pushing it away from the contact. A moment later, when the ball leaves the scratch, the surface will come back to its initial shape and the lubricant will be sucked back. The scratch would thus serve as a hydrodynamic pump, actively replenishing the contact with lubricant. The replenishment rate, would, of course, depend on the viscosity of the liquid. For PBOB (which has the lowest viscosity within the tested ionic liquids) at 100 °C the replenishment process is so efficient that tribofilm forming reactants are being washed away from the contact.

No significant improvement of the wear protective performance has been observed for ionic liquids applied on rougher SD and BW surfaces when compared to reference oils, at least according to the rather simple wear definition used in the top panels of Fig. 1, 5a,b. In fact, based on that definition, PBScB results in at least as much (if not more) wear than any of the reference oils (*vide infra*). It is important to note that whilst the dimensions of the wear scars in the cases where ionic liquids were used are similar to the reference oil cases, there are significant qualitative differences, with less ploughing and scarring, and more polishing. (In fact, the wear scar for PBMB on the BW surface could not be detected with the naked eye for the purposes of later ToF–SIMS analysis).

It is worth considering the PBScB case and the observed wear scar in particular. Immediately, the wear scar implies that any ionic lubricating layer formed is disrupted by the surface roughness (*cf.* Figs. 2, 5a,b). It would be expected that the rougher surfaces accelerate ionic liquid decomposition due to the energy released in asperity contacts. However, there is no significant generation of either a sacrificial tribofilm or particulate matter that might result in enhanced wear by physical means. As such, the enlarged wear scar relative to the Hertzian contact is likely due to chemical wear. Such chemical wear probably results from the breakdown of the anion of the ionic liquid to give boric acid and salicylic acid (Scheme 1b), which can react with the oxide layer on the surface^51^. Two comparisons must thus be explained: (i) why the PBScB wear scar is greater in the case of the rougher surfaces compared to the polished discs and (ii) why the PBScB wear scar is greater than the case for the other ionic liquids used. The first point is explained by greater breakdown of the PBScB when a rough surface is used; whilst this might be due to greater mechanical energy resulting in breakdown, this origin can be discounted given the fact that the same trend is not seen for all of the ionic liquids used. Rather, the difference might be explained by greater electrochemical breakdown as a result of increased charge development at the projections for the rougher surfaces^52^ as expected based on the fact that [BScB]^-^ is more readily oxidised than the other anions, with the aromatic system able to delocalise the unpaired electron^45,53^.

The second point—that the wear is greater for PBScB than the other ionic liquids—cannot be explained by abrasive wear given the lack of breakdown products observed. As such, dissolution of the iron oxide surface (chemical wear) is most likely. Whilst this outcome might simply be due to a greater amount of boric and organic acid being generated in these systems, the relative acidities of the organic acids generated might also contribute. Whilst oxalic acid is more acidic than salicylic acid in water^54,55^, the potential to effectively stabilise the anion through intramolecular hydrogen bonding in non-hydrogen bonding solvents inverts this acidity in non-protic solvents such as *N*,*N*-dimethylformamide^56^ and it is reasonable that this may be even further extended in ionic liquids such as these without any hydrogen bond donor sites.

**Figure 6:**
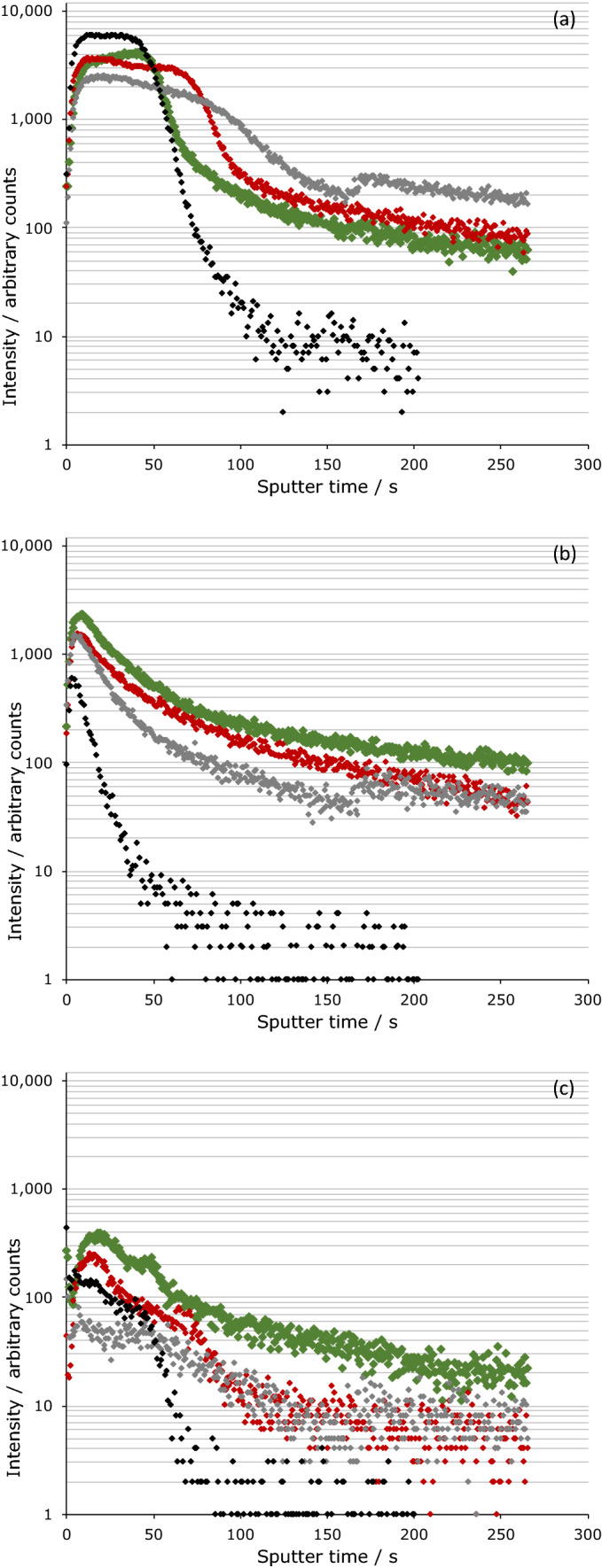
ToF–SIMS depth profiles for the BW surfaces, showing the change in ion intensity over time for **(a)** ^54^FeO_2_^−^, **(b)** BO^−^ and **(c)** Fe_2_B_3_O_3_^−^. The colours correspond to the ionic liquid used PBOB (green), and PBScB (red), with the untreated surface shown separately (grey). The untreated PD surface is also shown (black).

ToF–SIMS analysis performed on the BW samples for the two extreme cases, PBOB and PBScB (illustrated as depth profiles in Fig. 6), showed some key differences when compared to the PD samples (Fig. 3). The oxide layer in the bearing washers was significantly less defined than in the PD case, with lower initial intensities of the Fe_x_O_y_ signals (exemplified by the ^54^FeO_2_^−^ signal, Fig. 6a) which remained for a longer duration, only dropping to *ca.* 30 times the intensity of the corresponding signal for the polished steel; after the initial oxide layer there was still a significant amount of Fe_x_O_y_ signal present, at *ca.* 10 times the intensity of that in the polished steel (again, exemplified in Fig. 6a). This lack of definition is seen in all of the depth profiles of BW samples and is consistent with a rougher surface, including troughs (see Fig. 2, Supporting Information). The PBScB sample had intermediate behaviour, with the width of the initial oxide layer between that for the PBOB and the bearing washer, but the final signal intensities being comparable to the PBOB sample. These data further support that the surface is being polished during the experiment, with the PBOB ionic liquid resulting in the greatest extent of polishing.

The analysis of the boron containing signals shows a different pattern of signal intensities (Fig. 6b,c). As was the case for the Fe_x_O_y_ signals, the bearing washer case gave a much greater abundance of boron containing species than the polished disk. In contrast, the PBOB sample contains an even higher abundance than the bearing washer rather than similar levels to the polished disk. This result shows that while the surface is likely being polished, it is also being chemically changed and the ionic liquid is breaking down. This outcome is consistent with the previous analysis indicating that the ionic liquid is breaking down but suggests that the motion of the lubricant due to the scratches on the rough bearing washer surface is preventing the formation of a tribofilm. The PBScB sample again displays intermediate behaviour with the signal intensity being between that of the bearing washer and the PBOB sample.

Thus, there is a complex interplay between topography and chemical architecture. Firstly, roughness and topography need to be considered separately; while the presence of channels technically changes the roughness, the greater effect is in allowing replenishment of ionic liquid and transport of breakdown products away from the contact. The asperities themselves lead to alternative, electrochemical breakdown pathways that result in a different order of stability for the anions to that introduced above. Further, there is a fine balance between the formation of an effective lubricating layer and the extent of chemical wear.

## Conclusions

The work described herein has considered the effect of the structure of the orthoborate anion of phosphonium ionic liquids on their tribology. The varying halogen-free anion architecture in this series of ionic liquids was chosen to affect their intermolecular interactions, interfacial self-assembly properties and chemical stability, and hence their lubricating properties.

The orthoborate core was shown to be important in determining the chemical stability of the ionic liquids, with the size of the heteronuclear ring and the hybridisation of the constituent atoms being significant. The decoration of this ring, including incorporation of aryl components, is significant as it both introduces intermolecular interactions (with electronic and steric features) and affects the chemical stability of the anion.

ToF–SIMS analysis, in particular depth profiling, has been shown to be an effective method not only to investigate sacrificial tribofilm composition, but also to provide mechanistic information about lubrication, based on the analysis of thermal and thermomechanical chemical breakdown products.

All the ionic liquids displayed excellent friction and wear performance compared to non-ionic oils. The differences in lubricating performance between the ionic liquids could be directly tied to the chemical structure of the orthoborate anion. Notably, the breakdown of the anion is a precondition for the breakdown of the phosphonium cation; the necessary nucleophilic species are generated through the anion decomposition.

To exemplify, the ionic liquid with the smallest anion (and concomitantly most strained heteronuclear ring), PBOB, exhibits significant breakdown to give a lubricating hybrid tribofilm. The other ionic liquids do not break down to the same extent (likely due to reduced ring strain) and the salicylate derivative was most stable; in both of these cases the reduced friction observed suggesting the formation of a lubricating boundary layer with interactions involving the aryl groups.

Surface topography has a two-fold effect on the observed lubricating ability of the ionic liquids and the design of the lubricating system must take these demands into account. Firstly, the higher pressures and temperatures experienced in the asperity collisions lead to the breakdown even of the most chemically stable species. Secondly, coarse polishing introduces channels that enhance the viscosity-mediated transport of lubricant and breakdown products, both to and from the contact. Consequently, the surface topography must be also taken into account when considering the chemical transformations of the lubricant, as different topographies can introduce different mechanisms of decomposition. Thus, future formulations involving orthoborate ionic liquids are likely to combine advantageous architectural features via either mixing or a heteroleptic approach, and may extend to the use of these systems as components of oils and ionic greases.

Overall, this work has shown the importance of anion architecture and the tuning potential in the design of an ionic liquid lubricant to give the desired performance based on an understanding of their chemical stability and properties. More generally, lubricant design must adapt to the conditions for which the lubricant is to be used and this work demonstrates the manner in which a chemical understanding of the tribological outcome can be used to guide that design.

## Methods

The ionic liquids were prepared as described previously (PBMB and PBScB^40^, PBOB^57^) with spectroscopic and physical data matching that reported. Prior to use, these ionic liquids were dried under vacuum to constant weight and were found to be < 100 ppm water using Karl-Fischer titrimetry.

As benchmarks for the ionic liquid lubricating performance, three oils were used as references: two base stocks from ExxonMobil, mineral oil, CORE 2500, group 1 (MO)^58^; a poly-alpha-olefin oil cPAO100 (SO)^59^; and a fully formulated gear oil LOADWAY EP 220 from Statoil Lubricants, Sweden (FO)^60^. These oils were chosen such that their viscosity values also bridge the range of viscosities displayed by the ionic liquids. These values, for all lubricants tested, are shown in Table 1. These dynamic viscosity measurements were performed on a Discovery HR2 hybrid shearing rheometer (TA instruments) using a 40 mm plate geometry. Viscosity was measured at a constant temperature of 100 °C and a constant shear rate of 100 s^−1^. The temperature was controlled using a Peltier element situated below the lower plate. To decrease the heat flow through the upper plate, a thermal barrier insert was used.

**Table 1:** Viscosity of the ionic liquids and oils at 100 °C. The uncertainty reported is the measurement uncertainty of the instrument.

Liquid	Viscosity / mPa·s
PBOB	18.8 ± 0.1
PBMB	65.8 ± 0.1
PBScB	34.5 ± 0.1
SO	88.3 ± 0.1
MO	26.8 ± 0.1
FO	16.3 ± 0.1

### Test parameters and procedures

Tribological tests were performed on an SRV4 (Optimol Instruments Prüftechnik GmbH, Germany) test rig using a ball on plate configuration under ambient conditons. The tribological tests described herein were performed under oscillating/sliding conditions using a ball-on-plate configuration. This setup is common in the rapidly evolving field of ionic liquids lubrication^61–64^. A key reason for its popularity is that only a relatively small amount of material is required. This feature is important as it facilitates the testing of bespoke materials, creating an efficient feedback loop between testing and synthesis facilities.

Balls and plates were made of AISI 52100 bearing steel. Balls of 10 mm diameter were used as upper moving specimens in all tests. A new ball was used for each experiment. To study the effect of surface finish on lubrication, plates with three levels of surface finish were used; standard SRV disks (SD) with Ra0.037, SRV disks polished (PD) to Ra0.004 and sections of needle bearing washers (BW) as a representative of industrial grade surface with Ra0.032. Standard SRV disks were supplied by Optimol Instruments Prüftechnik GmbH, Germany. BW were obtained by cutting WS81105 bearing washers (NTN corporation, Japan). Surface topographies of the test surfaces in the study were recorded using a white light interferometer (ZYGO NewView). The measurements were performed using a 50 ×  zoom lens for at least three spots on each surface. The data were then processed using MountainsMap®8 software. The surface topography and bearing ratio curves are provided in the Supporting Information, Fig. S1.

Prior to the tests all steel samples were washed by sequential sonication in baths filled with acetone, isopropanol and ethanol, for at least 20 min with each solvent. After the last sonication step, surfaces were dried in the oven at 80 °C.

Fresh lubricant (*ca*. 50 μL) was placed on the plate surface at the beginning of each tribological test. A 20 N load was applied, then the plate surface was heated to the test temperature. The tribotest itself started after the temperature was found to be stable within ± 2 °C over a time interval of 5 min. All tests were performed at a temperature of 100 °C, with a stroke length of 1 mm and frequency of 50 Hz. Following the guidelines of ASTM D5707-19^65^, all tribotests were initiated by a 30 s running in phase at a lower load (20 N) than the test load (40 N). At the end of each test, lubricant from the upper (ball) and lower (plate) surfaces was collected and placed in a single plastic tube for further analysis.

Two types of tribological tests were performed (i) constant load (CL) tests with a constant load applied after the running in phase—these tests have been run at least three times with each lubricant for all three types of plates; and (ii) load carrying capacity (LCC) tests with load increased step-wise immediately after the running in up to 400 N (the step size was 50 N, step length was 5 min). Only data for the tests of PD type surfaces are presented for LCC tests and these have been run at least twice for each lubricant.

### Surface and lubricant condition analysis

After the tribotests, all plates were sonicated in chloroform and then in ethanol baths, to remove the test lubricant from the surface. The surfaces were then analysed using an Olympus PMG3 Inverted microscope. Wear was determined on the upper surfaces (balls) in terms of the wear scar diameter observed on the images (wear scars were approximated as circles). The PD surfaces were additionally analysed using a Jeol 7800F SEM (Scanning Electron Microscope) equipped with Bruker Quantax EDS (Energy Dispersive X-ray Spectroscopy) unit. SEM and EDS were performed at a beam voltage of 15 kV.

### ToF–SIMS analysis

Time-of-flight secondary ion mass spectrometry (ToF–SIMS) was performed using a TOF.SIMS 5 (IONTOF, Germany) at the Mark Wainwright Analytical Centre, University of New South Wales, on both ionic liquid samples and the solid surfaces. Liquid samples were placed as droplets onto silicon wafers prior to analysis. The primary analysis beam was Bi_3_^+^ clusters at 30 kV.

2D images were obtained covering a 500 μm square area and a resolution of 256 × 256 pixels, with 1 shot per pixel per frame. To produce a spectrum, a region of interest was defined containing the desired section and the pixels in this region integrated.

Depth profiles were obtained covering an 80 μm square area at a resolution of 64 × 64 pixels with two shots per pixel per frame. The sputtering beam was composed of caesium atoms ejected at 500 V with square cross-section 300 μm wide. The depth profiles were obtained using interlaced sputtering with one frame of imaging followed by eight frames of sputtering.

### Heat treatment of the ionic liquids

Each of the ionic liquids was heated using a heat gun (operating at *ca*. 400 °C). Heat treatments of 10 s and 60 s were applied. Visual inspection (based on colour and evolution of gas) indicated that breakdown of the [BOB]^−^ based ionic liquid was notably more rapid than the other two cases.

## Supplementary Information

Supplementary Information.
