# Supplementary material for: The effect of anion architecture on the lubrication chemistry of phosphonium orthoborate ionic liquids
Source: Sci Rep. 2021 Dec 15;11:24021. doi: 10.1038/s41598-021-02763-5 (PMC8674318; doi:10.1038/s41598-021-02763-5)
Supplement: Supplementary file 1 — Supplementary Information. [file 41598_2021_2763_MOESM1_ESM.docx]

**Electronic supporting information for**

“**The effect of anion architecture on the lubrication chemistry of phosphonium orthoborate ionic liquids**”

Bulat Munavirov, Jeffrey J. Black, Faiz Ullah Shah, Mark W. Rutland, Jason B. Harper and Sergei Glavatskih

**Table of contents**

[Surface topographies of the materials used 2](#_Toc85636929)

[Load carrying capacity tests 3](#_Toc85636930)

[Electron microscopy and X-ray spectroscopy of worn surfaces 5](#_Toc85636931)

[Averaged coefficients of friction and wear rates 6](#_Toc85636932)

[Time of Flight – Secondary Ion Mass Spectrometry analyses of worn surfaces 9](#_Toc85636933)

[Time of Flight – Secondary Ion Mass Spectrometry analyses of ionic liquids 12](#_Toc85636934)

[NMR spectra of ionic liquids before and after heating 14](#_Toc85636935)

[Mechanistic description of the breakdown of phosphonium orthoborate ionic liquids 21](#_Toc85636936)

[References 27](#_Toc85636937)

Surface topographies of the materials used


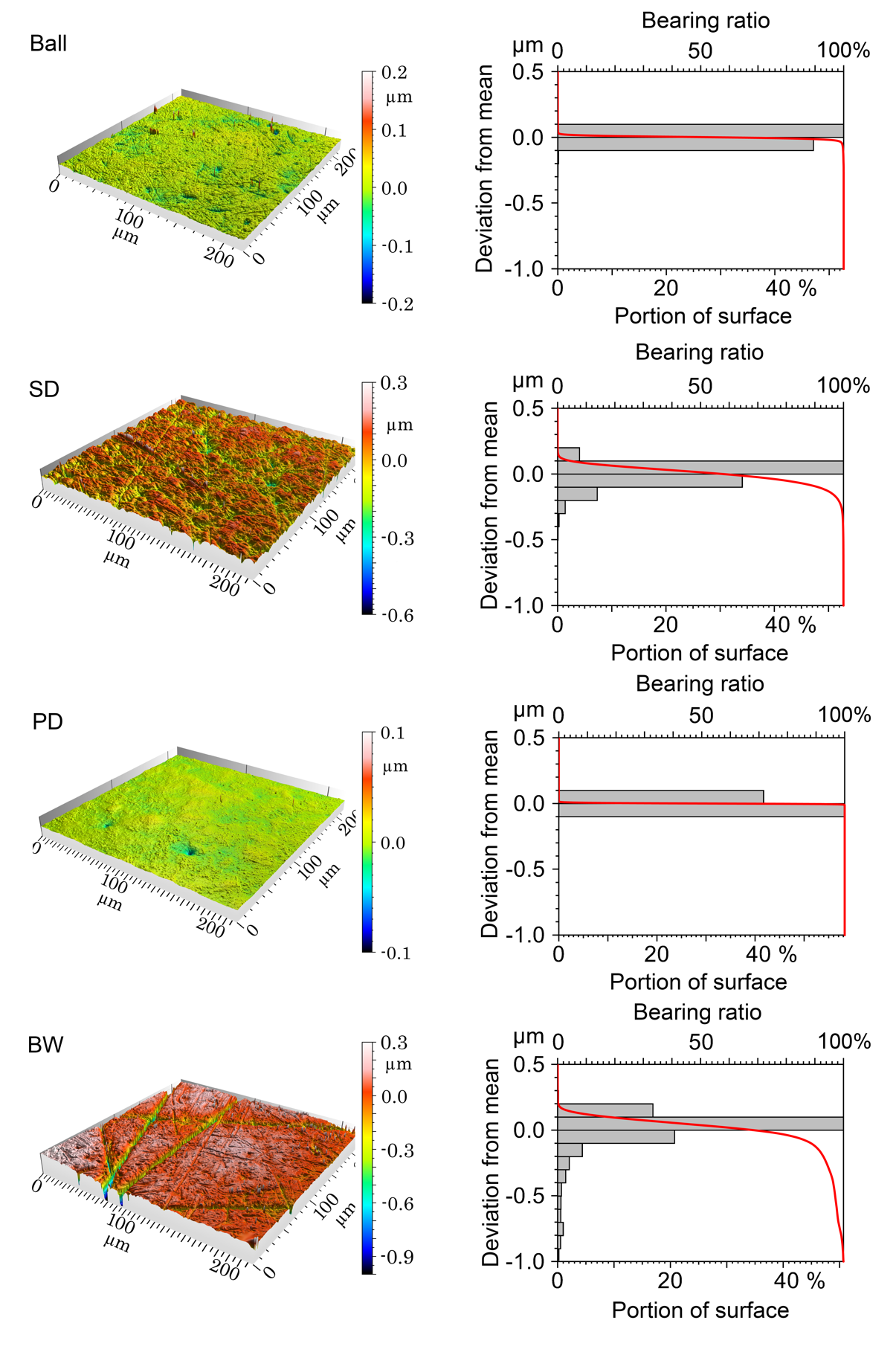


**Figure S1.** Surface topography, bearing ratio and asperity height distribution for the ball and the plates (standard disc (SD), polished disc (PD) and bearing washer (BW)) used in the tribological experiments.

Load carrying capacity tests

The durability of the tribofilms formed by PBOB, PBMB and PBScB was studied using increasing load conditions and these data are shown in Figure S2. In all cases the reference oils undergo a seizure event, as indicated by a dramatic increase in the friction coefficient on increasing load, which is maintained. In contrast, all ionic liquids tested sustain the increase in load; while for PBOB and PBScB there are short duration increases in friction (up to values >0.5) on increasing the load (indicating local scuffing events) and apart from these instantaneous spikes, the friction coefficients remain relatively constant. Such behaviour, according to ASTM D5706-16, indicates that seizure does not occur. PBOB clearly provides the most stable antiseizure performance compared to the other fluids indicating formation of the most robust tribofilms. The nature of this tribofilm is not immediately clear; a sacrificial tribofilm formed by breakdown is implicit from the above analysis while recent work^1^ indicates a lubricious ionic boundary layer. Thus, the most likely explanation is a hybrid tribofilm.

Interestingly, PBMB demonstrates a slightly different response towards an increase in load during the LCC tests; of particular note is that there are no friction spikes. Instead, PBMB demonstrates a distinct change of performance with load: at lower load values the friction coefficient changes very little and remains in the range *ca*. 0.07-0.08, then at an intermediate load range (250-300 N) there is a region where the friction coefficient rises slowly and then falls again. Finally, at higher loads the friction is again stable at values of *ca*. 0.1. The origin of these changes is not immediately clear, but it does suggest a change in the nature of the tribofilm. As argued above, a lubricating ionic boundary layer akin to that suggested in related work with PBOB under different conditions^1^ likely contributes significantly to the observed lubricating properties of PBMB. The load dependence as seen suggests that at some point the load is such that it can disrupt the boundary layer and change the lubricating properties of the system.


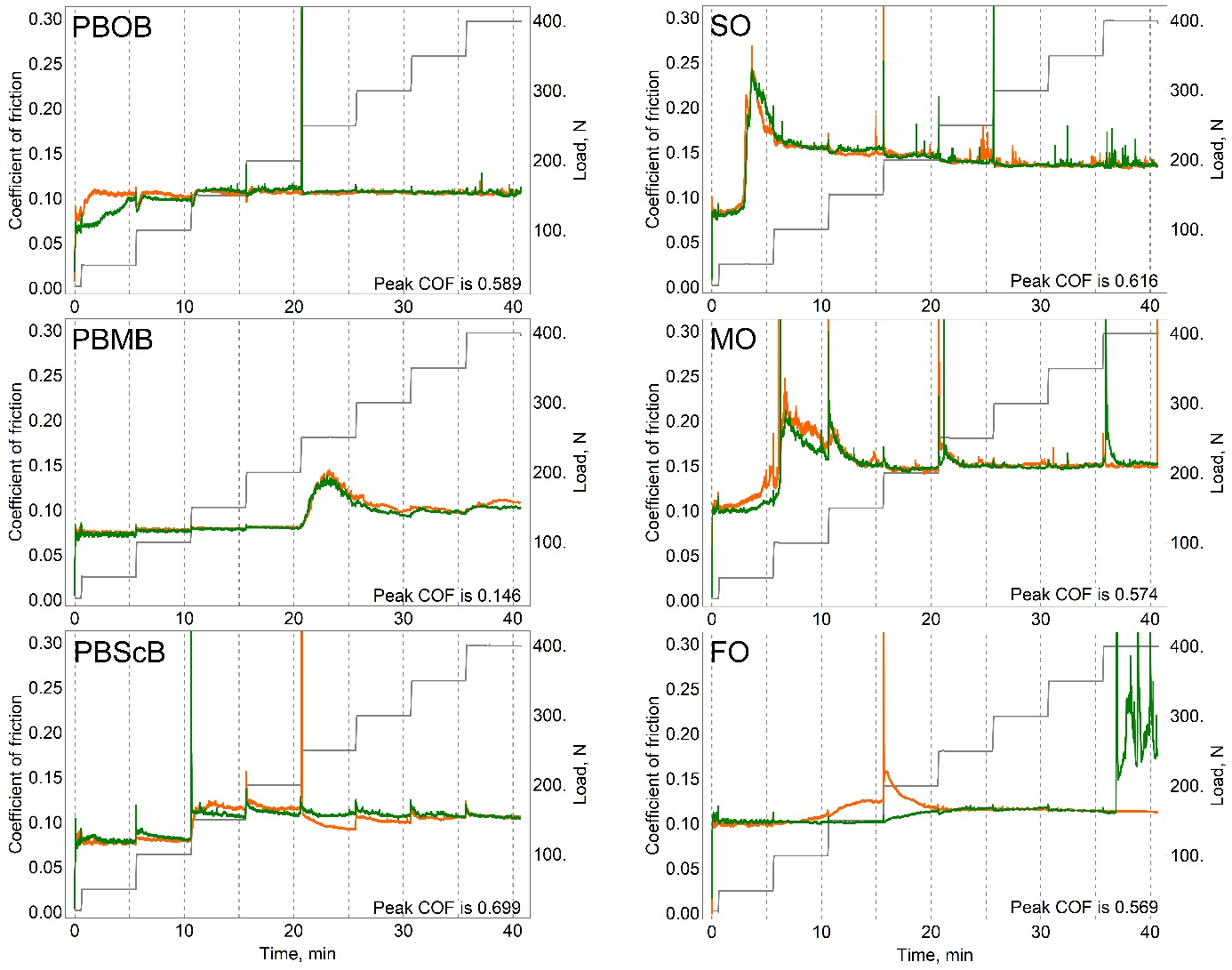

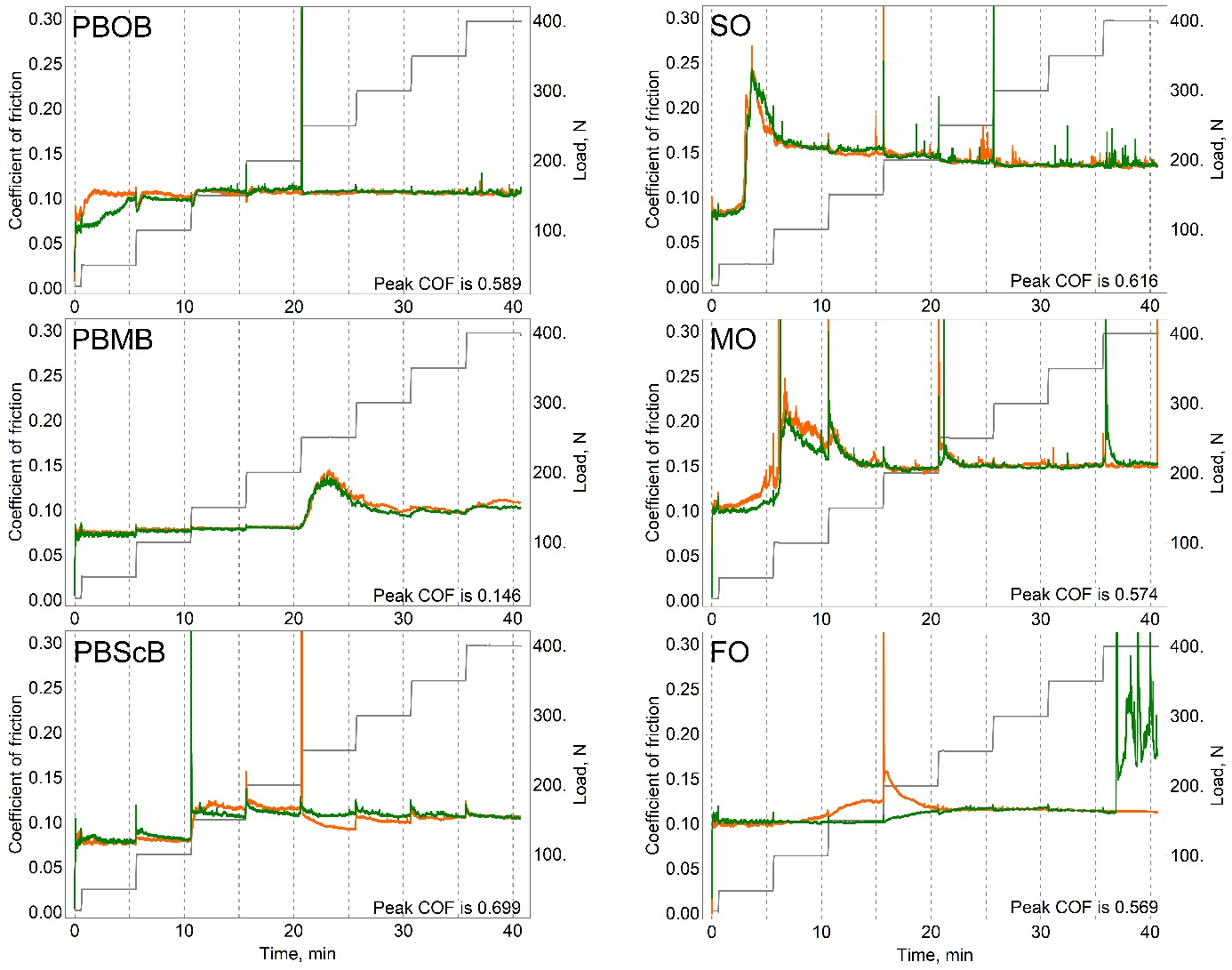


**Figure S2.** Variation in the friction coefficient with contact load (which varies stepwise as shown in gray) for systems containing the lubricants shown. The orange and green traces represent the outcomes of duplicate experiments.

Electron microscopy and X-ray spectroscopy of worn surfaces


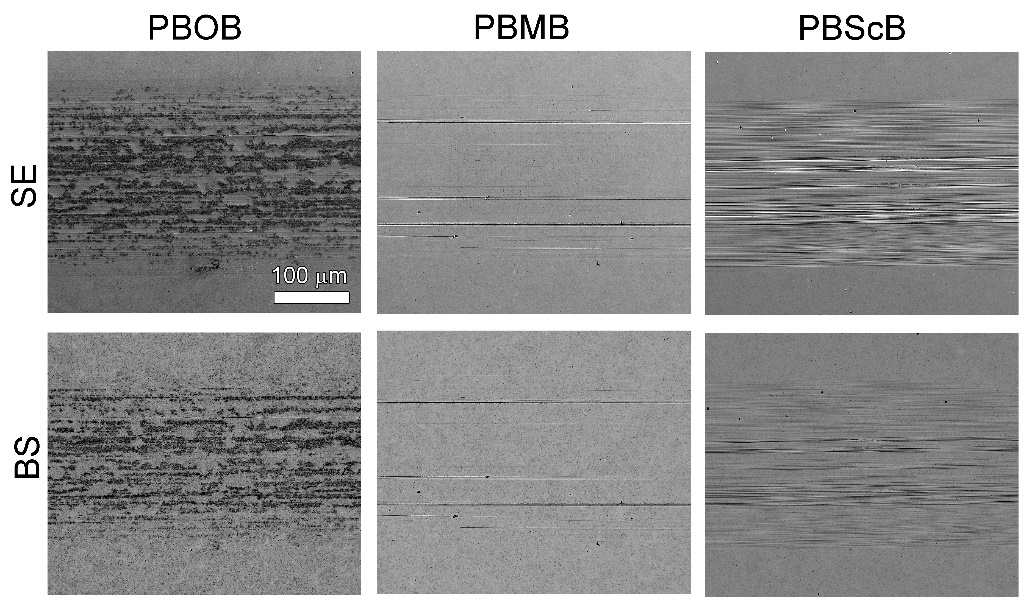


**Figure S3.** Scanning electron microscopy **(**SEM) images of the worn PD surfaces after tribological tests in the presence of one of the ionic liquids considered. The images are taken with the secondary (SE, top) and back scattered (BS, bottom) electrons.


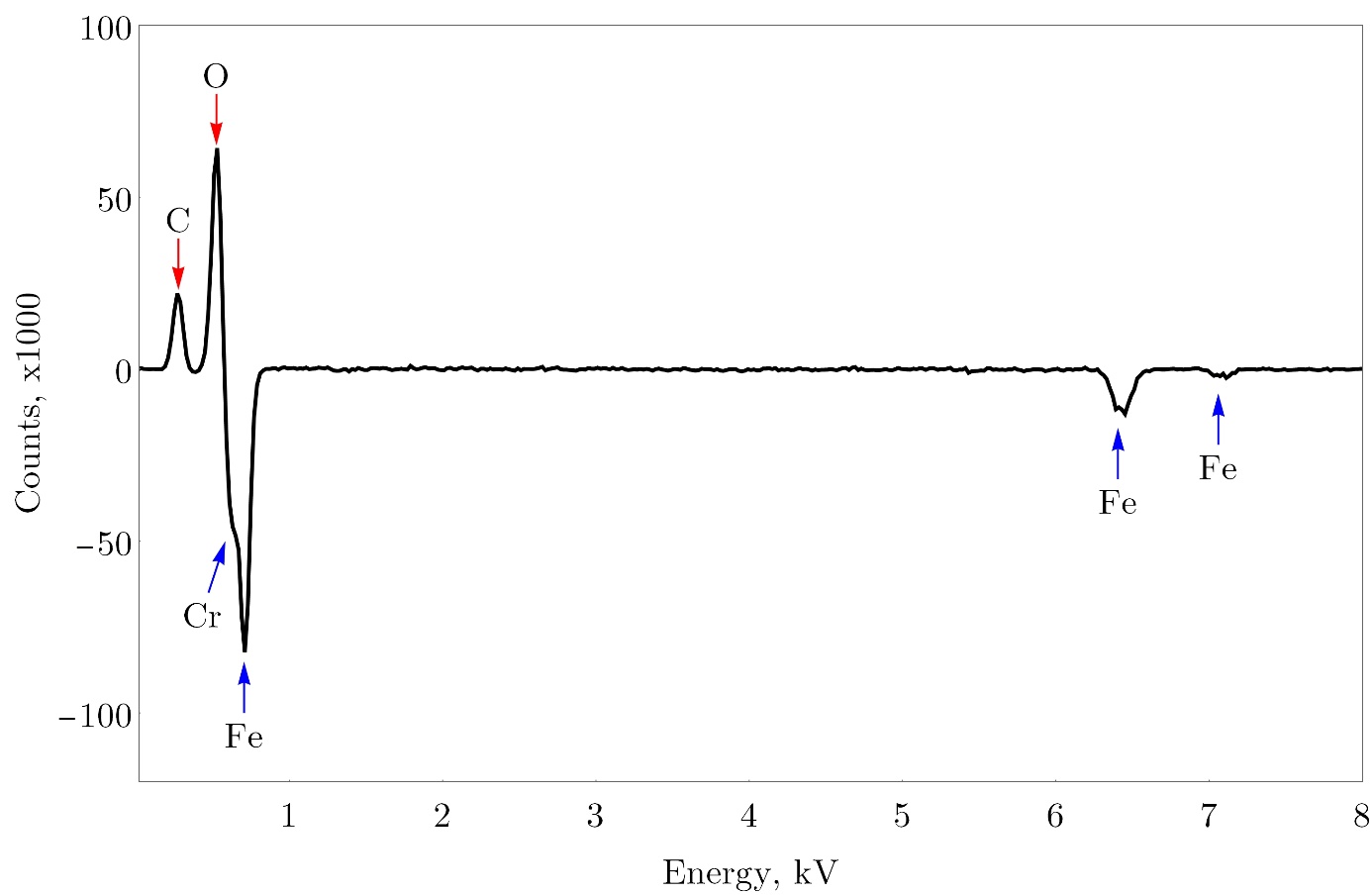


**Figure S4.** Differential Energy-dispersive X-ray spectra comparing results from the worn surface of a polished disc after lubrication experiments involving PBOB and the untreated polished disc.

Averaged coefficients of friction and wear rates

| **Lubricant** | **Average CoF** |
| --- | --- |
| PBOB | 0.102±0.004 |
| PBMB | 0.078±0.002 |
| PBScB | 0.082±0.003 |
| SO | 0.174±0.006 |
| MO | 0.158±0.003 |
| FO | 0.135±0.001 |

**Table S1.** Coefficients of friction averaged over the last ten minutes of the tribological tests on the polished discs for all repeats. Uncertainties reported are the standard deviation.

| **Lubricant** | **Average CoF** |
| --- | --- |
| PBOB | 0.109±0.010 |
| PBMB | 0.082±0.002 |
| PBScB | 0.067±0.003 |
| SO | 0.186±0.005 |
| MO | 0.166±0.006 |
| FO | 0.125±0.008 |

**Table S2.** Coefficients of friction averaged over the last ten minutes of the tribological tests on the standard discs for all repeats. Uncertainties reported are the standard deviation.

| **Lubricant** | **Average CoF** |
| --- | --- |
| PBOB | 0.081±0.004 |
| PBMB | 0.080±0.008 |
| PBScB | 0.061±0.006 |
| SO | 0.145±0.004 |
| MO | 0.141±0.010 |
| FO | 0.109±0.003 |

**Table S3.** Coefficients of friction averaged over the last ten minutes of the tribological tests on the bearing washers for all repeats. Uncertainties reported are the standard deviation.

| **Lubricant** | **Wear rate / 10^-7^ mm^3^ m^-1^** |
| --- | --- |
| PBOB | 1.0 |
| PBMB | 0.7 |
| PBScB | 0.8 |
| SO | 7.1 |
| MO | 5.7 |
| FO | 1.2 |

**Table S4.** Wear rates of balls averaged per unit distance for the tribological tests on the polished discs for all repeats. Uncertainties reported are the standard deviation.

| **Lubricant** | **Wear rate / 10^-7^ mm^3^ m^-1^** |
| --- | --- |
| PBOB | 1.3 |
| PBMB | 3.1 |
| PBScB | 9.4 |
| SO | 6.7 |
| MO | 5.0 |
| FO | 3.3 |

**Table S5.** Wear rates of balls averaged per unit distance for the tribological tests on the standard discs for all repeats. Uncertainties reported are the standard deviation.

| **Lubricant** | **Wear rate / 10^-7^ mm^3^ m^-1^** |
| --- | --- |
| PBOB | 1.1 |
| PBMB | 3.6 |
| PBScB | 9.2 |
| SO | 8.0 |
| MO | 2.4 |
| FO | 1.9 |

**Table S6.** Wear rates of balls averaged per unit distance for the tribological tests on the bearing washers for all repeats. Uncertainties reported are the standard deviation.

| **Lubricant** | **Wear rate / 10^-8^ mm^3^ s^-1^** |
| --- | --- |
| PBOB | 9.9 |
| PBMB | 6.8 |
| PBScB | 7.9 |
| SO | 71.4 |
| MO | 56.7 |
| FO | 12.1 |

**Table S7.** Wear rates of balls averaged per unit time for the tribological tests on the polished discs for all repeats. Uncertainties reported are the standard deviation.

| **Lubricant** | **Wear rate / 10^-8^ mm^3^ s^-1^** |
| --- | --- |
| PBOB | 13.0 |
| PBMB | 30.6 |
| PBScB | 93.9 |
| SO | 66.7 |
| MO | 49.6 |
| FO | 33.3 |

**Table S8.** Wear rates of balls averaged per unit time for the tribological tests on the standard discs for all repeats. Uncertainties reported are the standard deviation.

| **Lubricant** | **Wear rate / 10^-8^ mm^3^ s^-1^** |
| --- | --- |
| PBOB | 11.3 |
| PBMB | 35.5 |
| PBScB | 91.5 |
| SO | 80.3 |
| MO | 24.0 |
| FO | 18.6 |

**Table S9.** Wear rates of balls averaged per unit distance for the tribological tests on the bearing washers for all repeats. Uncertainties reported are the standard deviation.

Time of Flight – Secondary Ion Mass Spectrometry analyses of worn surfaces

**Figure S5.** Time of Flight – Secondary Ion Mass Spectrometry (ToF-SIMS) depth profiles for the PD surfaces, showing the change in ion intensity over time for a series of ions involving carbon and oxygen (a) C_2_O^-^ and (b) CO_2_^-^. The colours correspond to the ionic liquid used PBOB (green), PBMB (blue) and PBScB (red), with the untreated surface shown separately where appropriate (black).

**Figure S6.** Time of Flight – Secondary Ion Mass Spectrometry (ToF-SIMS) depth profiles for the PD surfaces, showing the change in ion intensity over time for a series of ions involving oxygen (a) O^-^, (b) O_2_^-^ and (c) OH^-^. The colours correspond to the ionic liquid used PBOB (green), PBMB (blue) and PBScB (red), with the untreated surface shown separately where appropriate (black).

**Figure S7.** Time of Flight – Secondary Ion Mass Spectrometry (ToF-SIMS) depth profiles for the PD surfaces, showing the change in ion intensity over time for a series of ions involving phosphorus (a) PO_2_^-^ and (b) PO_3_^-^. The colours correspond to the ionic liquid used PBOB (green), PBMB (blue) and PBScB (red), with the untreated surface shown separately (black).

Time of Flight – Secondary Ion Mass Spectrometry analyses of ionic liquids

**Figure S8.** Relative abundance (absolute) of selected ions in the ToF-SIMS analysis of the pristine ionic liquid (blue), the ionic liquid sample heated for *ca.* 10 s (orange), the ionic liquid sample heated for *ca.* 60 s (grey) and the ionic liquid after use in tribological tests (yellow) for PBOB. From left to right, the ions are (i) [BOB]^-^, (ii) BO_2_^-^, (iii) C_2_BO_5_^-^ and (iv) C_2_HO_4_^-^.

**Figure S9.** Relative abundance (absolute) of selected ions in the ToF-SIMS analysis of the pristine ionic liquid (blue), the ionic liquid sample heated for *ca.* 10 s (orange), the ionic liquid sample heated for *ca.* 60 s (grey) and the ionic liquid after use in tribological tests (yellow) for PBMB. From left to right, the ions are (i) [BMB]^-^, (ii) BO_2_^-^, (iii) C_8_H_7_O_3_^-^ and (iv) C_8_H_6_BO_4_^-^.

**Figure S10.** Relative abundance (absolute) of selected ions in the ToF-SIMS analysis of the pristine ionic liquid (blue), the ionic liquid sample heated for *ca.* 10 s (orange), the ionic liquid sample heated for *ca.* 60 s (grey) and the ionic liquid after use in tribological tests (yellow) for PBScB. From left to right, the ions are (i) [BScB]^-^, (ii) BO_2_^-^, (iii) C_7_H_5_O_3_^-^ and (iv) C_7_H_4_BO_4_^-^.

NMR spectra of ionic liquids before and after heating

**Figure S11.** ^1^H NMR spectrum (400 MHz, DMSO-*d*_6_) of a sample of the ionic liquid PBOB before (bottom) and after (top) having been heated at *ca*. 400 °C for 10 s. (Bottom: *δ* 0.83‑0.91 (m, 12H, -CH_3_), 1.22-1.52 (m, 38H), 2.08‑2.20 (m, 8H, P-CH_2_); Top: Note additional signal *δ* 1.50-1.60 (m).)

**Figure S12.** ^11^B NMR spectrum (128 MHz, DMSO-*d*_6_) of a sample of the ionic liquid PBOB before (bottom) and after (top) having been heated at *ca*. 400 °C for 10 s. (Bottom: *δ* 7.36 (s); Top: Note additional signals *δ* 5.09 (br s), 8.45 (s).)

**Figure S13.** ^13^C NMR spectrum (101 MHz, DMSO-*d*_6_) of a sample of the ionic liquid PBOB before (bottom) and after (top) having been heated at *ca*. 400 °C for 10 s. (Bottom: *δ* 14.3, 14.5, 17.6, 17.7, 18.2, 20.95, 21.0, 22.3, 22.6, 28.5, 29.1, 29.2, 29.4, 29.5, 29.52, 29.54, 30.1, 30.3, 30.8, 31.7; Top: Note additional signals *δ* 14.4, 18.1, 20.8, 21.65, 21.7, 22.4, 27.5, 28.2, 30.4, 30.5, 30.7, 31.3.)

**Figure S14.** ^31^P{^1^H} NMR spectrum (162 MHz, DMSO-*d*_6_) of a sample of the ionic liquid PBOB before (bottom) and after (top) having been heated at *ca*. 400 °C for 10 s. (Bottom: *δ* 33.77; Top: Note additional signals *δ* 37.80, 46.00.)

**Figure S15.** ^1^H NMR spectrum (400 MHz, DMSO-*d*_6_) of a sample of the ionic liquid PBMB before (bottom) and after having been heated at *ca*. 400 °C for either 10 s (middle) or 60 s (top). (Bottom: *δ* 5.10-5.18 (3 x s, 2H, -CH-), 7.24-7.29 (m, 1H, ArH), 7.30-7.40 (m, 2H, ArH), 7.43-7.55 (m, 2H, ArH); Middle: Note additional/increased signals *δ* 7.94 (d, *J* = 7.6 Hz); Top: Note additional/increased signals *δ* 4.85 (s), 6.80 (d, *J* = 8.0 Hz), 7.20-7.60 (m).) Note that multiple isomers are possible.

**Figure S16.** ^11^B NMR spectrum (128 MHz, DMSO-*d*_6_) of a sample of the ionic liquid PBMB before (bottom) and after (top) having been heated at *ca*. 400 °C for either 10 s (middle) or 60 s (top). (Bottom: *δ* 11.0 (s); Top: Note additional signals *δ* 6.5 (s), 20.2 (br s).)

**Figure S17.** ^13^C NMR spectrum (101 MHz, DMSO-*d*_6_) of a sample of the ionic liquid PBMB before (bottom) and after (top) having been heated at *ca*. 400 °C for either 10 s (middle) or 60 s (top). (Bottom: *δ* 126.51, 126.54, 126.58, 126.61, 127.6, 127.65, 128.35, 128.4, 129.7, 140.95, 141.0, 141.1; Middle: Note additional signal *δ* 128.7; Top: Note additional signals *δ* 126.2, 126.7, 126.9, 127.1, 128.0, 128.1, 128.75, 128.85, 128.95, 128.97, 129.1; 137.5, 142.3)

**Figure S18.** ^31^P{^1^H} NMR spectrum (162 MHz, DMSO-*d*_6_) of a sample of the ionic liquid PBMB before (bottom) and after (top) having been heated at *ca*. 400 °C for either 10 s (middle) or 60 s (top). (Bottom: *δ* 33.78; Top: Note additional signal *δ* 46.02.)

**Figure S19.** ^1^H NMR spectrum (400 MHz, DMSO-*d*_6_) of a sample of the ionic liquid PBScB before (bottom) and after (top) having been heated at *ca*. 400 °C for either 10 s (middle) or 60 s (top). (Bottom: *δ* 5.10-5.18 (m, 1H, ArH), 6.88(td, *J =* 7.6, 1.6 Hz, 1H, ArH), 7.45 (td, *J =* 7.6, 1.6 Hz, 1H, ArH), 7.74 (dd, *J* = 7.6, 1.6 Hz, 1H, ArH); Middle/Top: Note additional signals *δ* 6.73-6.79 (m), 7.12-7.20 (m).). Note that multiple isomers are possible.

**Figure S20.** ^11^B NMR spectrum (128 MHz, DMSO-*d*_6_) of a sample of the ionic liquid PBScB before (bottom) and after (top) having been heated at *ca*. 400 °C for either 10 s (middle) or 60 s (top). (Bottom: *δ* 4.0 (s); Top: Note additional signal *δ* 20.0 (br s).)

**Figure S21.** ^13^C NMR spectrum (101 MHz, DMSO-*d*_6_) of a sample of the ionic liquid PBScB before (bottom) and after (top) having been heated at *ca*. 400 °C for either 10 s (middle) or 60 s (top). (Bottom: *δ* 116.1, 117.2, 118.0, 118.2, 119.0, 129.9, 130.4, 134.6, 135.0, 159.5, 162.5, 164.3, 172.3; Middle/Top: Note additional signals *δ* 116.0, 129.7, 157.8). Note that signals are *lost* on heating.

**Figure S22.** ^31^P{^1^H} NMR spectrum (162 MHz, DMSO-*d*_6_) of a sample of the ionic liquid PBScB before (bottom) and after (top) having been heated at *ca*. 400 °C for either 10 s (middle) or 60 s (top). (Bottom: *δ* 32.3, 34.0, 37.1, 46.1; Top: Note additional signal *δ* 26.3, 49.5, 50.2, 58.0.)

Mechanistic description of the breakdown of phosphonium orthoborate ionic liquids

Throughout the work described here, ToF-SIMS data were analysed based on predicted breakdown products; it is reasonable to consider this in terms of the mechanistic pathways introduced in Table S10 below. It should be noted that the chemistry behind these is uncontroversial and has even been introduced previously to explain the importance of impurities in the stability of phosphonium-based ionic liquids.^2^

For the cation of the ionic liquid (and resultant breakdown products), there are several well‑described mechanisms; while others might be included (such as radical processes), the ones listed are considered most likely. [These mechanisms may apply in either the analysis method (ToF-SIMS) or the treatment methods (wear tests, heating). In the context here, it is assumed that the treatment methods are being considered. Any breakdown in the analysis method is taken into account by considering the pristine ionic liquid samples.] Importantly, these mechanisms predict cleavage of the phosphorus‑carbon bonds, oxygenation of the phosphorus centre and ultimately the formation of phosphoric acid (and associated deprotonated species). For the anions, the principle result is loss of the chelating ligand, eventually generating boric acid (and deprotonated forms, noting the potential for polymerisation of boric acid/borates); see the descriptions in Table S11 below. While reactions of the ligand are possible, the effect of their reaction and simple loss are the same.

The heat treatment and tribological evaluation of the ionic liquids demonstrates the relative stability of the ionic liquids used. The mechanisms proposed below explain the breakdown of the ionic liquids and can be applied to either heating or tribofilm formation. However, the experiments above raise two particular questions.

1. There is greater breakdown of the [BOB] anion *cf.* the other anions considered. Why is this the case?
2. The breakdown of the cation of the ionic liquid is not independent of the anion; in all of the cases considered, a common cation was used but the breakdown was different. What is the mechanistic origin of such?

The first point is covered broadly in the main text, but the latter is more mechanistic so is covered here.

If it is taken that the [BOB] anion breaks down to a greater extent than the other anions, why might this affect the breakdown of the cation? Consider the mechanisms in Table S11; the bulk of them require a nucleophile/base. In the breakdown process of [BOB], boric acid (and related acidic species are formed). Either this species could act as the nucleophile/base or, more likely, the deprotonated form (borate) does – the deprotonated form is generated from reaction, most likely with iron oxide (which is basic); exemplars are shown in Table S12. [This process also generates water – it can be said to 'regenerate' it, given it is needed in the first stage of the breakdown of the anion. Irrespective, the water generated can react further.] The boronate products can further break down, either through reaction with other nucleophiles in the case of substitution, or reaction with a base in the case of the protonated form. Irrespective, the result is that increased breakdown of the cation as a result of the breakdown products of the anion. [Once formed, phosphoric acid could deprotonate and react in the same way (hence speeding the breakdown of the cation too). However, this occurs *after* the initial effect of the boron derivatives.]

*Table S10: Mechanisms of breakdown - cation*

| **Name** | **Mechanism**  [Bases and nucleophiles are arbitrarily shown with a negative charge. Neutral species are also appropriate (*e.g.* water) if less reactive.] | **Likely fate of products of reaction** | **Other points** |
| --- | --- | --- | --- |
| Elimination (Hofmann) |  | Phosphorus species reacts further. Likely oxidation.  Alkene species either washed away or volatile under conditions of analysis.  Protonated base either washed away or volatile under conditions of analysis. | While drawn for the phosphonium system, also applies for other phosphorus species (extents may vary)  Nature of the base needs to be considered (adventitious water, halide, breakdown product from anion). Could also be surface of material. |
| Substitution  (reverse Menschutkin) |  | Phosphorus species reacts further. Likely oxidation.  Alkane species either washed away or volatile under the conditions of the analyses. | While drawn for the phosphonium system, also applies for other phosphorus species (extents may vary)  Nature of the nucleophile needs to be considered (adventitious water, halide, breakdown product from anion). Could also be surface of material. |
| Elimination  (to give phosphorane)  [In principle, a similar substitution process could also occur (see above) but the outcome would be the same. Simple deprotonation might occur but would effectively be reversible.] |  | Phosphonium ylides (also known as phosphoranes) are extremely basic and likely protonates (to give phosphonium). May act as nucleophile but proton transfer probable.  Alkene species either washed away or volatile under conditions of analysis.  Protonated base either washed away or volatile under conditions of analysis. | Really only likely for phosphonium systems.  Nature of the base needs to be considered (adventitious water, halide, breakdown product from anion). Could also be surface of material. |
| Oxidation |  | Phosphorus species likely react further (oxidation, elimination, substitution).  Oxygen is assumed to come from air. | Mechanism is autoxidation - a radical process and multistep (not shown). May be favoured by the presence of the metal (source of electrons). |
| Elimination  (O- leaving group)  [In principle, a similar substitution process could also occur (see above) but the outcome would be the same.] |  | Phosphorus species could be shown as protonated or deprotonated form.  Phosphorus species could react further (oxidation, breakdown).  Alkene species either washed away or volatile under conditions of analysis.  Protonated base either washed away or volatile under conditions of analysis. | Ultimately this results in phosphate/phosphoric acid. May react with metal oxide layer.  Nature of the base needs to be considered (adventitious water, halide, breakdown product from anion). Could also be surface of material. |

*Table S11: Mechanisms of breakdown - anion*

| **Name** | **Mechanism**  [Bases and nucleophiles are arbitrarily shown with a negative charge. Neutral species are also appropriate (*e.g.* water) if less reactive.] | **Likely fate of products of reaction** | **Other points** |
| --- | --- | --- | --- |
| Hydrolysis |  | Boron species could be shown as protonated or deprotonated form.  Boron species may polymerise.  Organic species either washed away or volatile under conditions of analysis. | Applies to all systems though likelihood dependent on ring strain and stability of oxyanion.  Ultimately this results in borate / boric acid. Latter reacts with metal oxide surface.  Does require water (though in principle, other nucleophile can also interact). |
| Reduction |  | May lose ligand and polymerise (see below). Any doubly reduced species very reactive.  May react with water. | May occur but has functionally the same outcome as simple hydrolysis.  Which boron species will favour these?  Probably not much difference (electron goes to boron) - bond cleavage will be favoured by stability of the anion that forms; the carboxylates are all comparable. (If anything BOB faster than BMB faster than BScB) |
| Oxidation |  | Result of oxidation is likely breakdown of the ligand. Resultant boron products follow same pathways indicated elsewhere. Organic products will breakdown further, but definitely be volatile and hence lost. | It is unlikely that initial loss of the electron happens from B - all evidence suggests that this happens on the ligand.  Likewise, not all ligands would stabilise the radical cation to the same extent. Aromatic systems will do so. Calculated data has salicylate being easiest and borate hardest – expect mandelate in the middle. |
| Polymerisation  (borate) |  | - | Generates water.  Drawn as dimerization, in principle can repeat. May also have three dimensional element.  Functionally polymerisation may be difficult to distinguish from clustering of equivalent borate. |
| Polymerisation  (other) |  | Would expect these borates would break down, particularly losing organic component.  Water or other ligand lost during washing or analysis. | Generates water.  Drawn as dimerization, in principle can repeat. May also have three dimensional element.  Functionally polymerisation may be difficult to distinguish from clustering of equivalent borate. |
| Breakdown of organic component |  | Likely to break down further, expect to lose organic component.  While gases here, other breakdown products certainly volatile. | Products shown for loss of carbon monoxide then carbon dioxide from oxalate. Will vary with nature of side chain.  Unlikely to have impact as organic species generally lost. |

*Table S12: Mechanisms of breakdown – invoking borate to breakdown the cation of the ionic liquid.*

| **Name** | **Mechanism** | **Likely fate of products of reaction** |
| --- | --- | --- |
| Elimination (Hofmann) |  | Phosphorus species reacts further. Likely oxidation.  Alkene species either washed away or volatile under conditions of analysis.  Boronate reacts with base (likely iron oxide) |
| Substitution  (reverse Menschutkin) |  | Phosphorus species reacts further. Likely oxidation.  Alkane species either washed away or volatile under the conditions of the analyses. But potentially reacts further (with other nucleophiles, including simply water). |

References

1 Rohlmann, P. *et al.* Boundary lubricity of phosphonium bisoxalatoborate ionic liquids. *Tribol. Int.* **161**, 107075, doi:10.1016/j.triboint.2021.107075 (2021).

2 Deferm, C. *et al.* Thermal stability of trihexyl(tetradecyl)phosphonium chloride. *Phys. Chem. Chem. Phys.* **20**, 2444-2456, doi:10.1039/c7cp08556g (2018).
